# Progress in the elimination of hepatitis C virus infection: A population-based cohort study in Spain

**DOI:** 10.1371/journal.pone.0208554

**Published:** 2018-12-04

**Authors:** Regina Juanbeltz, Alejandra Pérez-García, Aitziber Aguinaga, Iván Martínez-Baz, Itziar Casado, Cristina Burgui, Silvia Goñi-Esarte, Jesús Repáraz, José Manuel Zozaya, Ramón San Miguel, Carmen Ezpeleta, Jesús Castilla, EIPT-VHC Study Group

**Affiliations:** 1 Instituto de Salud Pública de Navarra—IdiSNA, Pamplona, Spain; 2 CIBER Epidemiología y Salud Pública, Pamplona, Spain; 3 Department of Clinical Microbiology, Complejo Hospitalario de Navarra—IdiSNA, Pamplona, Spain; 4 Department of Pharmacy, Complejo Hospitalario de Navarra—IdiSNA, Pamplona, Spain; 5 Department of Gastroenterology and Hepatology, Complejo Hospitalario de Navarra, Pamplona, Spain; 6 Department of Internal Medicine, Complejo Hospitalario de Navarra—IdiSNA, Pamplona, Spain; Centers for Disease Control and Prevention, UNITED STATES

## Abstract

**Background:**

The World Health Organization set targets to eliminate hepatitis C virus (HCV) infection through detection and treatment of all cases by 2030. This study aimed to describe the progress and difficulties in the elimination of HCV infection in Navarra, Spain.

**Methods:**

Using electronic healthcare databases, we performed a population-based prospective cohort study to describe changes in the prevalence of diagnosed active HCV infection at the beginning of 2015 and the end of 2017, the rate of new diagnoses and the rate of post-treatment viral clearance (PTVC) during this period.

**Results:**

At the beginning of 2015 there were 1503 patients diagnosed with positive HCV-RNA, 2.4 per 1000 inhabitants, and at the end of 2017 the prevalence had decreased by 47%. In the study period, 333 (18 per 100,000 person-years) new positive HCV-RNA cases were detected, but only 76 (23%; 4.2 per 100,000 person-years) did not have anti-HCV antibodies previously detected. Prevalent cases and new diagnoses of active infection were more frequent in men, people born in 1950–1979, HIV-infected patients and in those with lower income levels. Among patients with HCV-RNA, 984 achieved PTVC (22.7 per 100 person-years). PTVC was less frequent in patients born before 1940, in immigrants and in patients with lower income levels.

**Conclusions:**

The prevalence of diagnosed active HCV infection has dropped by almost half over three years, because the number of patients with PTVC was much higher than the number of new diagnoses. Interventions specifically targeted at population groups with less favourable trends may be necessary.

## Introduction

In the European Union about 3 million people were chronically infected with hepatitis C virus (HCV) by 2015 [1], and Spain was one of the most affected countries [2–4]. Without treatment 55–85% of HCV infected persons may develop chronic hepatitis [5,6]. Direct-acting antivirals (DAAs) have demonstrated high efficacy, achieving sustained virological response in most patients [7–11].

In 2016 the World Health Organization set targets to eliminate HCV infection as a major public health threat by 2030 [12]. These targets should be translated into operational objectives in geographical areas and population groups, including the detection of undiagnosed HCV-infections, diagnosis of active infections among HCV positive patients and antiviral treatment of active infections [13, 14]. In 2015, the National Health System in Spain launched a plan to progressively provide access of all HCV-infected patients to the DAAs regimens [15], without treatment restrictions since early 2017. In Spain a high proportion of active HCV infections have already been diagnosed. Some infections may have an incomplete diagnosis, if the detection of anti-HCV has not been followed by determination of the HCV-RNA, making it difficult to establish whether active infection continues or has cleared spontaneously. Finally, some active infections may remain undiagnosed [16].

Good practices on aspect related to HCV-elimination have been described [17,18], but comprehensive studies evaluating the progress towards the HCV-elimination are necessary. In order to monitor the process of eliminating HCV infection in Spain, we quantified the changes in the prevalence of diagnosed active HCV infection, the rate of new diagnoses and the rate of post-treatment viral clearance (PTVC) according to socio-demographic characteristics of the population during the first three years when DAAs were widely used. The rate of PTVC was considered as a summarized indicator of the access, adherence and effectiveness of antiviral treatment.

## Materials and methods

### Study population and design

The study population included people with stable residence in the Navarra region, Spain, and covered by the Regional Health Service (about 615,000 inhabitants). Using electronic healthcare databases we performed a prospective cohort study to describe and compare the prevalence of HCV-RNA positive diagnosed cases at the beginning of 2015 and at the end of 2017. We also monitored new HCV-RNA diagnoses and the PTVC during this follow-up period.

### Information sources and variables

Registered results of HCV antibodies (anti-HCV) and of HCV-RNA were available from microbiology databases that include all the test results in laboratories of the Navarra Health Service since the introduction of these tests in clinical practice. During the study period about 4% of the population was annually tested for anti-HCV in Navarra with an increased screening effort over time. Some of these tests were done in persons with suspected HCV infection, and others were done within screening protocols (risk exposure history, prison inmates, pregnant women, blood donors, etc.). The HCV diagnoses included serology for anti-HCV (ARCHITECT, Abbott Laboratories, Wiesbaden, Germany), and RT-PCR for HCV-RNA (Cobas HCV, Roche Diagnostics, Mannheim, Germany) in all patients with a positive anti-HCV result.

A prevalent active HCV infection was defined as HCV-RNA detection in the most recent test previous to 1st January 2015. Patients testing positive for HCV-RNA for the first time between 1st January 2015 and 31st December 2017 were considered new diagnoses of active HCV infection regardless of when they could have acquired the HCV infection. For some analyses we considered those patients with a first positive result for both anti-HCV and HCV-RNA during the study period.

The information on HIV infection was obtained from the regional enhanced surveillance system [19]. From the databases of hospital pharmacies we obtained the HCV treatments administered up to the end of 2017. The guidelines of the European Association for the Study of the Liver panel and the Spanish health authorities were followed in the treatment of patients [20]. Since 2015, oral DAAs have been widely used and replaced interferon containing treatments. In HCV-RNA positive patients, viral clearance was defined as undetectable HCV-RNA in the last test during the study period. We defined PTVC as undetectable HCV-RNA in all tests performed after the antiviral therapy, and considered it the endpoint indicator of a process that summarizes early access, good adherence and high effectiveness of the DAAs therapies among the patients who initially had an active HCV infection.

From administrative databases we obtained the sex, birth year, origin country (native or immigrant), municipality of residence and income level. Place of residence in municipalities with more than 10,000 inhabitants was considered urban and all others were considered rural. The income level was established in the categories used by the Spanish Health System: low (no incomes or great dependence), lower-middle (<18,000 €/year) and high income level (>18,000 €/year).

### Statistical analysis

The prevalence of HCV-RNA positive tests in the population was calculated at the beginning of 2015 and the end of 2017, and the percentage of change between the two estimates was obtained. The chi-square test was used to compare percentages. Multivariate logistic regression models were used to identify differences in the prevalence, in terms of adjusted odds ratios (ORs) with their 95% confidence intervals (CIs).

Among the initially susceptible population we calculated the rate of new HCV-RNA detection between 2015 and 2017. We also calculated the rate of new diagnoses of positive anti-HCV and HCV-RNA. Among the population with a diagnosis of active HCV infection at the beginning or during the follow-up, we obtained the incidence rate of PTVC during the period 2015–2017. The factors associated with the rate of new diagnoses of active HCV infection and with the rate of PTVC were analysed using Poisson regression models to estimate the adjusted rate ratio (RR) and its 95% CI. Multivariate analyses were adjusted by sex, birth decade, origin country, place of residence, income level, HIV infection and year of diagnosis or PTVC.

### Ethical considerations

The study protocol was approved by the Navarra Ethical Committee for Clinical Research. The data were obtained from epidemiological surveillance and electronic healthcare databases and were analyzed anonymously.

## Results

### Prevalence of diagnosed active HCV infection

On first January 2015 there were 1503 patients with a HCV-RNA positive result in their last determination (2.4 per 1000 inhabitants). The prevalence was almost double in men (3.1) than in women (1.6; p<0.001). The highest prevalence was observed in people born in the 1960s (8.4 per 1000 inhabitants), which included more than half of all cases (52%). Birth cohorts born in the 1950s and 1970s also exceeded 2 cases per 1000 inhabitants, and only in birth cohorts born after 1980 the prevalence was below 1 per 1000 inhabitants. About 78% of cases had lower-middle or low income level, and they showed the highest prevalence, 2.5 and 11.1 per 1000 inhabitants, respectively. Among people infected with HIV the prevalence of active HCV infection was 30% and among people with active HCV infection the prevalence of coinfection reached 19% (Table 1).

**Table 1:** Prevalence of diagnosed active infection by hepatitis C virus in Navarra, Spain, on 1st January 2015 and 31st December 2017, and percentage of change between the two dates, according to socio-demographic characteristics.

	1st January 2015			31st December 2017			% of change	
	Cases	Rate x 1000	*P* value	Cases	Rate x 1000	*P* value	%	*P* value
**Sex**			<0.001			<0.001		
Men	952	3.1		474	1.6		-50	<0.001
Women	551	1.6		313	1.0		-39	<0.001
**Birth cohort**			<0.001			<0.001		
Before 1930	24	1.3		18	1.4		10	0.922
1930–1939	75	1.9		61	1.9		-10	0.802
1940–1949	106	1.9		55	1.0		-47	<0.001
1950–1959	232	3.2		99	1.4		-57	<0.001
1960–1969	789	8.4		376	4.0		-52	<0.001
1970–1979	212	2.1		134	1.3		-36	<0.001
1980–1989	42	0.6		33	0.4		-20	0.324
1990–1999	10	0.2		4	0.1		-60	0.106
2000 or after	13	0.1		7	0.1		-50	0.176
**Origin country**			0.205			0.100		
Native	1346	2.5		669	1.3		-50	<0.001
Immigrant	157	2.0		118	1.5		-24	0.022
**Place of residence**			<0.001			0.020		
Rural	495	2.0		282	1.2		-42	<0.001
Urban	1008	2.7		505	1.4		-50	<0.001
**Income level**			<0.001			<0.001		
High	327	1.4		122	0.5		-64	<0.001
Lower-middle	906	2.5		485	1.4		-44	<0.001
Low	270	11.1		180	6.2		-44	<0.001
**HIV infection**			<0.001			<0.001		
No	1214	2.0		681	1.1		-44	<0.001
Yes	289	300.1		106	104.6		-65	<0.001
**Total**	1503	2.4		787	1.3		-47	<0.001

In the multivariate analysis, the prevalence of positive HCV-RNA in 2015 was lower in women than in men (OR = 0.6, 95% CI 0.5–0.6), and was lower in immigrants than in the native population (OR = 0.5, 95% CI 0.4–0.6), while the factors associated with higher prevalence were HIV infection (OR = 89.0, 95% CI 75.3–105.1), birth before 1990, urban residence (OR = 1.4, 95% CI 1.3–1.6) and lower-middle (OR = 2.1, 95% CI 1.8–2.4) and low income level (OR = 8.2, 95% CI 6.9–9.8) (Table 2).

**Table 2:** Variables associated with the prevalence of diagnosed active hepatitis C virus infection on 1st January 2015 in Navarra, Spain.

	Adjusted odds ratio^a^	95% confidence interval	*P* value
**Sex**			
Men	Reference		
Women	0.6	0.5–0.6	<0.001
**Birth cohort**			
Before 1930	8.5	4.1–17.8	<0.001
1930–1939	12.0	6.2–23.2	<0.001
1940–1949	12.0	6.3–22.9	<0.001
1950–1959	18.3	9.7–34.5	<0.001
1960–1969	40.5	21.7–75.7	<0.001
1970–1979	11.3	6.0–21.2	<0.001
1980–1989	3.1	1.6–6.2	0.001
1990–1999	Reference		
2000 or after	0.7	0.3–1.7	0.486
**Origin country**			
Native	Reference		
Immigrant	0.5	0.4–0.6	<0.001
**Place of residence**			
Rural	Reference		
Urban	1.4	1.3–1.6	<0.001
**Income level**			
High	Reference		
Lower-middle	2.1	1.8–2.4	<0.001
Low	8.2	6.9–9.8	<0.001
**HIV infection**			
No	Reference		
Yes	89.0	75.3–105.1	<0.001

^a^ Logistic regression model including all the variables listed in the table.

**Fig 1:**
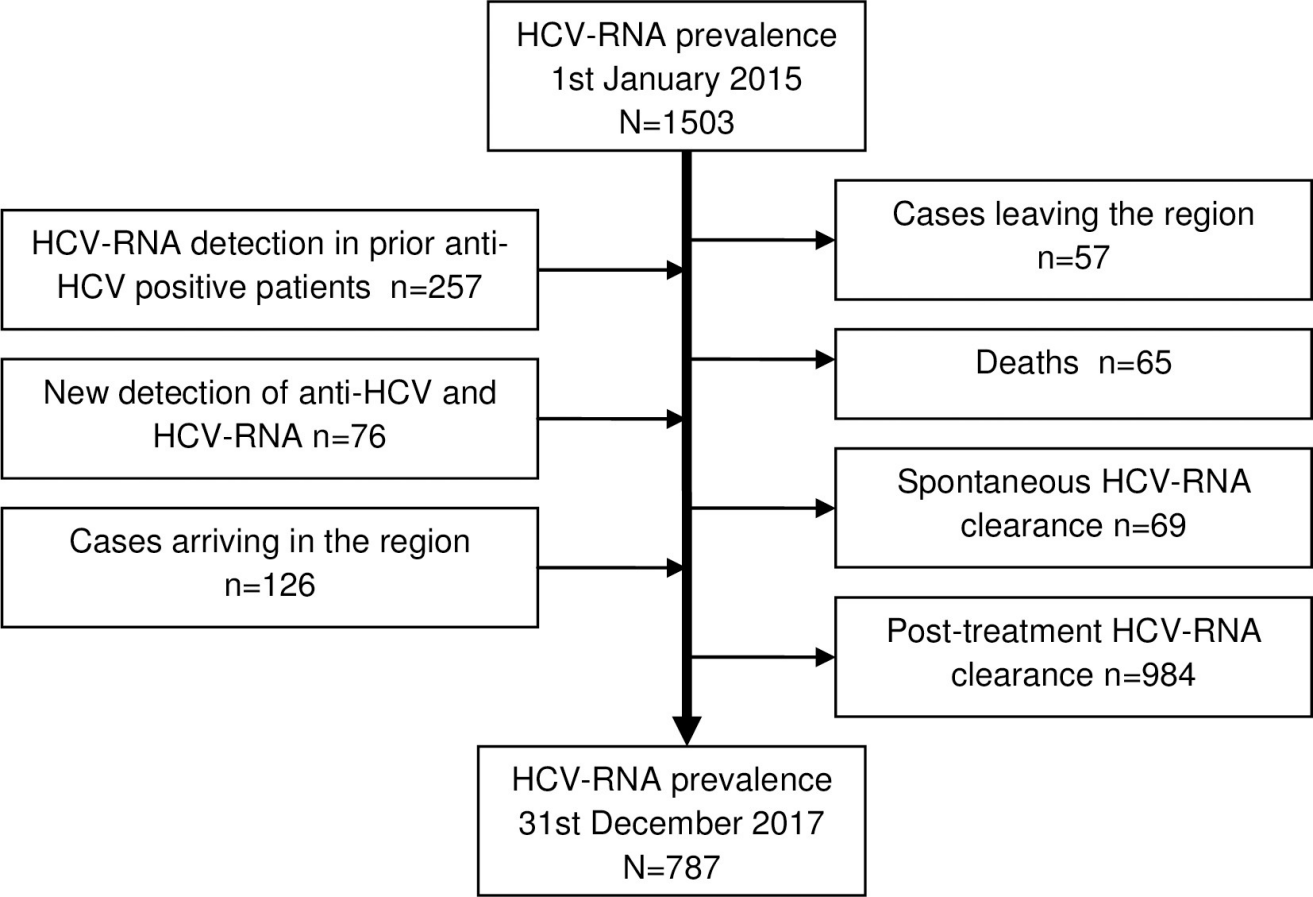
Input and output flows in the population-based cohort study, Navarra, Spain, 2015–2017.

From the beginning of 2015 until the end of 2017, active HCV infection was detected in 257 patients who had been diagnosed with anti-HCV in previous years, 76 persons were newly diagnosed with anti-HCV and HCV-RNA, and 126 patients previously diagnosed with active HCV infection arrived in the region during the study period. On the other hand, 57 active HCV-infected patients left the region, 65 died, 69 became spontaneously HCV-RNA negative, and 984 had completed the treatment and became HCV-RNA negative (Fig 1).

As a result of that, the prevalence of diagnosed HCV-RNA declined by 47%, from 2.4 to 1.3 cases per 1000 inhabitants (p<0.001). Decreases in the prevalence of HCV-RNA were observed among almost all population groups, but with different magnitude. The decline in males was 50% and in females 39%. The prevalence decreased around 50% in the cohorts born between 1940 and 1969, but non-statistically significant declines were observed in cohorts born before 1940 and after 1980. The decline in the prevalence of active HCV infection was more pronounced in the native population (50% vs. 24%), in HIV-coinfected patients (65% vs. 44%), and in the high income level category (Table 1).

### New diagnoses of active HCV infection

From 2015 to 2017 a total of 333 patients were detected with active HCV infection for the first time (18.5 per 100,000 person-years); the majority of them (n = 257; 77%) had tested positive for anti-HCV antibodies in previous years, but had not been tested for HCV-RNA. In general, the predictors of new diagnoses of active HCV infection were the same as those described for the prevalence of active HCV infection; furthermore, the rate of new diagnoses of HCV-RNA presented a statistically significant decline in 2017 as compared to 2015 (RR = 0.7, 95% CI 0.6–1.0) (Table 3).

**Table 3:** New diagnoses of RNA positive for hepatitis C virus regardless of the time since positive antibody detection by socio-demographic characteristics. Navarra, Spain, 2015–2017.

	New diagnoses	Rate per 100,000 person-years	Adjusted rate ratio^a^	95% confidence interval	*P* value
**Sex**					
Men	224	25.1	Reference		
Women	109	12.0	0.4	0.3–0.5	<0.001
**Birth cohort**					
Before 1930	3	6.3	12.2	1.3–117.2	0.031
1930–1939	24	21.7	40.8	5.5–302.3	<0.001
1940–1949	21	12.6	26.1	3.6–199.5	0.001
1950–1959	51	24.0	47.7	6.6–345.2	<0.001
1960–1969	163	59.5	111.0	15.5–792.4	<0.001
1970–1979	56	18.5	36.1	5.0–260.7	<0.001
1980–1989	14	6.5	11.4	1.5–87.0	0.019
1990–1999	1	0.6	Reference		
2000 or after	0	0	0	NA	0.999
**Origin country**					
Native	288	18.3	Reference		
Immigrant	45	19.9	0.6	0.5–0.9	0.008
**Place of residence**					
Rural	137	18.8	Reference		
Urban	196	18.3	1.1	0.9–1.3	0.516
**Income level**					
High	56	8.1	Reference		
Lower-middle	214	20.6	3.3	2.5–4.5	<0.001
Low	63	82.8	13.3	9.1–19.3	<0.001
**HIV infection**					
No	318	17.7	Reference		
Yes	15	806.5	15.4	9.1–26.1	<0.001
**Year of diagnosis**					
2015	121	20.3	Reference		
2016	124	20.7	1.0	0.8–1.3	0.826
2017	88	14.5	0.7	0.6–1.0	0.027
**Total**	333	18.5			

^a^ Poisson regression model including all the variables listed in the table.

NA, not available.

In 2015, 2016 and 2017, anti-HCV tests were performed in 25,555, 25,383 and 28,499 patients, respectively, in Navarra (4 per 100 person-year). However, only 76 patients were newly diagnosed with a positive result for both anti-HCV and HCV-RNA (4.2 per 100,000 person-years). The rate of new diagnoses of positive anti-HCV and HCV-RNA was 8 per 100,000 person-years or higher in HIV-infected people, in the population born between 1950 and 1969, and in those with low income level. There was only one case in persons born after 1990. In the multivariate analysis, a new diagnosis of anti-HCV and HCV-RNA was less frequent in women (RR = 0.5, 95% CI 0.3–0.9), and was more frequent in the population infected with HIV (RR = 22.1, 95% CI 7.9–61.8), in those born between 1950 and 1969, and in those with lower-middle (RR = 3.6, 95% CI 1.9–6.8) or low income level (RR = 8.4; 95% CI, 3.5–20.0) (Table 4).

**Table 4:** New diagnoses of antibodies and positive RNA results for hepatitis C virus by socio-demographic characteristics. Navarra, Spain, 2015–2017.

	New diagnoses	Rate per 100,000 person-years	Adjusted rate ratio^a^	95% confidence interval	*P* value
**Sex**					
Men	47	5.3	Reference		
Women	29	3.2	0.5	0.3–0.9	0.010
**Birth cohort**					
Before 1930	2	4.2	7.9	0.7–87.6	0.093
1930–1939	5	4.5	8.4	1.0–72.0	0.053
1940–1949	1	0.6	1.3	0.1–20.3	0.867
1950–1959	17	8.0	16.3	2.2–122.5	0.007
1960–1969	32	11.7	22.3	3.0–163.8	0.002
1970–1979	11	3.6	7.0	0.9–54.6	0.062
1980–1989	7	3.3	5.6	0.7–45.6	0.107
1990–1999	1	0.6	Reference		
2000 or after	0	0.0	0	NA	0.999
**Origin country**					
Native	64	4.1	Reference		
Immigrant	12	5.3	0.8	0.4–1.6	0.559
**Place of residence**					
Rural	31	4.2	Reference		
Urban	45	4.2	1.1	0.7–1.7	0.713
**Income level**					
High	12	1.7	Reference		
Lower-middle	54	5.2	3.6	1.9–6.8	<0.001
Low	10	13.1	8.4	3.5–20.0	<0.001
**HIV infection**					
No	72	4.0	Reference		
Yes	4	215.1	22.1	7.9–61.8	<0.001
**Year of diagnosis**					
2015	23	3.9	Reference		
2016	28	4.7	1.2	0.7–2.1	0.465
2017	25	4.1	1.1	0.6–1.9	0.731
**Total**	76	4.2			

^a^ Poisson regression model including all the variables listed in the table.

NA, not available.

### Post-treatment viral clearance

During the follow-up period, a total of 1962 patients diagnosed with active HCV infection accounted for 4329 person-years and 1053 cases of viral clearance (24.3 per 100 person-years). Viral clearance was spontaneous in 69 patients (1.6 per 100 person-years) and occurred in 984 patients who had completed the antiviral treatment. The rate of PTVC was 22.7 per 100 person-years, with no differences by sex, place of residence, birth cohort or HIV infection. The only exception was people born before 1940 in whom the rate of PTVC was lower (RR = 0.3, 95% CI 0.1–1.0). PTVC was less frequent in immigrants than in the native population (RR = 0.7, 95% CI 0.6–0.9), and among the population with lower-middle (RR = 0.8, 95% CI 0.7–0.9) or low income level (RR = 0.7, 95% CI 0.5–0.8). The rate of PTVC increased to 29.7 per 100 person-years in 2017 (RR = 1.5, 95% CI 1.3–1.8) (Table 5).

**Table 5:** Rate of post-treatment viral clearance among patients with diagnosis of active hepatitis C infection by socio-demographic variables. Navarra, Spain, 2015–2017.

	Person-years with active infection	Post-treatment viral clearance	Rate per 100 person-years	Adjusted rate ratio^a^	95% confidence interval	*P* value
**Sex**						
Men	2672	647	24.2	Reference		
Women	1657	337	20.3	0.9	0.8–1.1	0.213
**Birth cohort**						
Before 1940	321	18	5.6	0.3	0.1–1.0	0.045
1940–1949	293	65	22.2	1.3	0.5–3.6	0.601
1950–1959	634	189	29.8	1.8	0.7–4.8	0.250
1960–1969	2199	548	24.9	1.5	0.6–4.0	0.427
1970–1979	671	130	19.4	1.2	0.5–3.3	0.681
1980–1989	151	28	18.5	1.3	0.5–3.7	0.606
1990–1999	26	4	15.4	Reference		
2000 or after	34	2	5.9	0.4	0.1–2.0	0.257
**Origin country**						
Native	3815	898	23.5	Reference		
Immigrant	514	86	16.7	0.7	0.6–0.9	0.008
**Place of residence**						
Rural	1470	321	21.8	Reference		
Urban	2859	663	23.2	1.0	0.9–1.2	0.706
**Income level**						
High	853	246	28.8	Reference		
Lower-middle	2642	571	21.6	0.8	0.7–0.9	<0.001
Low	834	167	20.0	0.7	0.5–0.8	<0.001
**HIV infection**						
No	3573	788	22.1	Reference		
Yes	756	196	25.9	1.1	0.9–1.3	0.182
**Year**						
2015	1681	337	20.0	Reference		
2016	1442	289	20.0	1.0	0.9–1.2	0.758
2017	1206	358	29.7	1.5	1.3–1.8	<0.001
**Total**	4329	984	22.7			

^a^ Poisson regression model including all the variables listed in the table.

## Discussion

In 2015, when the use of the second generation of DAAs was expanded in Spain, the prevalence of diagnosed active HCV infection in Navarra was high and showed important socio-demographic differences. In the following three years this prevalence has decreased to near half, thanks to the large number of patients with PTVC. An important reduction in the prevalence was observed in all population groups, although the socio-demographic inequalities have not disappeared.

The rate of diagnoses of active HCV infection was 18 per 100,000 person-years, mainly as result of the first HCV-RNA testing in patients diagnosed with anti-HCV years ago. The decline in this rate of diagnoses of active infection in 2017 suggests a progressive reduction in the number of patients with positive anti-HCV who were pending HCV-RNA testing. During the period 2015–2017 a considerable proportion of the population was tested for anti-HCV, but only 4.2 new diagnoses of anti-HCV with active infection per 100,000 person-years were detected. This is consistent with the moderate rate of undiagnosed HCV-infections that has been described in Navarra [16].

Males and people born between 1950 and 1979 presented the highest prevalence rates of active HCV infection by both, the beginning and the end of the study period, and they also showed the highest rates of new diagnoses of active infection. This distribution is consistent with the pattern of the epidemic of injecting heroin consumption in Spain, which was responsible for many of these infections [21,22]. It is also consistent with the recently reported pattern of transmission among men who have sex with men [23], and is not very different from the distribution of HCV infection described in other countries [24,25].

The prevalence of active infection was also high among the people born before 1950, who accounts for most cases of iatrogenic HCV transmission [3]. The rate of PTVC was significantly lower among people born before 1940. Although since early 2017 there is no restriction in the DAAs treatments in Spain, advanced age and the presence of comorbidities may reduce the priority of treatment in practice; therefore, some interventions aimed at improving the recruitment of these populations to be treated may be necessary.

A recent meta-analysis reported no major differences in the prevalence of anti-HCV among native and immigrant populations in Spain [26]. Our results describe a lower prevalence and rate of new diagnoses of active HCV infection in the immigrant population after adjustment for other socio-demographic characteristics. The high transmission rate during the past century in Spain may explain this finding [2]. In immigrants, a worse rate of PTVC was observed, which indicates late request of medical care, since all the study population had free health care. The higher prevalence of active HCV infection in urban residents is consistent with other studies, and may be due to the higher frequency of risk behaviour for HCV transmission in cities [23].

Independently of other variables, lower income levels were associated with a higher prevalence and rate of new HCV-RNA diagnoses, as well as a lower rate of PTVC. Similar findings were described in the interferon era and the progressive reduction of differences during the DAAs era [27], and other studies have found that HIV-coinfected patients with lower income were less likely to initiate treatment [28,29]. In our study the prevalence of active HCV infection declined less in the lower income level categories so that the pre-existing socio-demographic inequalities in the prevalence have remained. The association between income level and active HCV infection may have several explanations: some risk exposures for HCV infection, especially injecting drug use, were more frequent in the low socio-economic level; the manifestations of HCV chronic infection may cause occupational difficulties and a resulting decline in income level; mental problems or drug addiction may favour the HCV infection as well as a decline in income level; and lastly, income level may be associated with late request for medical care and worse compliance with treatment, even in countries where HCV treatment is free of charge for the patient [29,30].

HIV infection was associated with a much higher prevalence and rate of new diagnoses of active HCV infection, which is consistent with results reported by other authors [23]. However, no differences were observed in the rate of PTVC by HIV status, which may be due to the specific priority of HIV-infected patients for antiviral treatments [15].

Several limitations may affect these results. Undiagnosed active HCV infections would be the main limitation; however, in spite of the high number of analysed persons only 76 new diagnoses of active infection were detected in three years, which suggests that the number of undiagnosed infections is probably low [16]. The fact that some patients with positive anti-HCV had not been tested for HCV-RNA may have resulted in an underestimate of the prevalence of active HCV infections; however these cases decreased during the study period. We analysed the factors associated with PTVC, but spontaneous clearance was also observed in few patients [31]. We considered PTVC the change from detectable to undetectable HCV-RNA in all available tests after the treatment as a summarized indicator of access, adherence and effectiveness of antiviral treatment. This concept does not exactly match the definition of sustained virological response usually used in clinical trials or clinical studies as a treatment effectiveness outcome [32].

The situation described is rapidly changing, thus some results may have short-term validity and updates may be necessary. Since interventions have been introduced in Navarra to improve the hepatitis C control program, the observed differences may decline in subsequent years. Other factors, such as differential mortality and migrations may affect the prevalence of active HCV infection. The high mobility of some population groups makes them difficult to study, although the inputs and outputs of the population partially offset each other. Although the epidemiology of HCV infection and the introduction of DAAs have been relatively homogeneous in Spain, we can not rule out some geographical differences.

## Conclusions

In a comprehensive population-based approach, a favourable trend in the prevalence of active HCV infection has been observed in Spain, with a reduction by nearly half after three years of use of DAAs. This may be explained by the fact that the rate of viral clearance has been much higher than the rate of new diagnoses. Even in a country with universal healthcare and HCV treatment free of charge for the patient, we found that lower income levels were associated with a higher prevalence, higher rate of new diagnosis of active HCV infection and a lower frequency of PTVC. Immigrants and older population also showed a lower rate of PTVC. These findings suggest the need of interventions specifically targeted at population groups with less favourable trends.
